# Address on Medicine

**Published:** 1888-05

**Authors:** John Cronyn

**Affiliations:** President State Medical Association, Professor Principles and Practice of Medicine and Clinical Medicine, Niagara University


### *ADDRESS ON MEDICINE.**

By JOHN CRONYN, M. D.

President State Medical Association, Professor Principles and Practice of Medicine and Clinical Medicine, Niagara University.

"As agriculture, to those who are in health, holds out the expectation of aliment, so medicine promises to the sick a recovery from disease:" Thus wrote Celsus 1800 years ago, and so may it be written to-day, with this difference, that in both a more enlightened measure is used to determine their success. The *gourmand* requires his dishes delicious and many, the fulness of his desires being satisfied only in the filling of his stomach. The *gourmet*, more dainty, delicate, and capricious in his longings, makes his demands with equal assurance that he, too, will be gratified: and why not? Do we not see the hillside, the valley, and the fallow made tributary to their wants? The plough, the harrow, and the roller are to service put. The

*Read before the N. Y. State Medical Association.

trench, the furrow, or the ditch is used, as best may accord with finest production of the food required. And when too frequent use of the once virgin soil necessitates a dressing, like an ill-conditioned ulcer, behold, at once, the fertilizer there: its fruit is forthwith seen, agriculture its promise keeps, and health moves on through ailment.

Does medicine do as well? If not, why? To give a history of the progress of medicine—adopting the chronological order into which it is most commonly divided, viz., from the earliest records we possess to the decline of Roman literature, from this period to the revival of letters, and from the introduction of the inductive philosophy to the present time, would no doubt, to many, be exceedingly interesting, if not profitable. It will be sufficient for my purpose, however, to but succinctly refer to a few of the characteristics of the earliest periods, and deal more in detail with subjects nearer our own time.

Mentioning the history of the science in its earliest records, it will suffice to remark that in proportion to the progress of civilization, greater and more active means were being constantly invented for the cure or alleviation of diseases, or for the repair of injuries to which the body is ever prone. These were directed almost entirely by the observance of such consequences as appeared from natural causes, or were due to certain kinds of food causing an elimination of such peccant substances as were supposed to produce disease. And so, too, with regard to injuries, for at this early period the value of excluding the wounded part from the air, and of the application of graduated pressure, was recognized; and these means were used to serve their purposes as now, and might be called the ancient method of antisepsis. In France, Germany, Italy and Great Britain, men of genius and learning were not wanting who put forth theory after theory, principle upon principle, and experience surmounted by observation. Under the ægis of one or the other leader were disciples in great numbers, who in their turn lived but to upset the doctrines of their masters, and, announcing something new, to fall like the others before the tide of advancing knowledge in philosophy and inductive reasoning, coupled, however, at all times with that ever-present antagonism of opinion, begotten of hatred and envy, to be found nowhere else to the same degree as in the profession of medicine. He who may find time to consider, after the manner of the historian, the changing features of opinion and doctrine I have indicated, will discover that here and there those subjects of greatest utility as a basis of correct practice were found most prominent in the theories and speculations of this or that writer—his reasoning, experience, and observation having always in view "the promise to the sick that their disease should be removed."

Coming to our own times, the conflicting opinions long in vogue embarrass and retard; for, unlike every other branch of science, our actual information does not increase at all proportionately to our experience; and, although truth is not to be attained in any science without much labor and research, yet, for those willing to bestow time and attention, it is, for the most part, attainable; or if, in the investigation, it elude our grasp, the reason is manifest. In other sciences, too, when we enter upon an inquiry, or propose to ourselves any definite object of experiment or observation, we are able to say whether the result has been satisfactory, whether the end sought was reached. Not so in medicine, for there are many peculiarities necessarily connected with the subject which render it extremely difficult to appreciate the value of experiment and observation. Can anything better elucidate this than the changing doctrines and practice of olden time, to which I have before alluded, and the progressive changes due to that want of certitude, or common consent to the results of experiment, even in our own day? But a few years ago, the whole medical world was divided in opinion as to whether the human constitution had undergone a complete transformation, or disease had changed its type, the basis of opinion being observation, experiment, and the experience of actual practice. The most potent agent in the therapeutics of our forefathers—bleeding—was no longer thought compatible with the cure of disease; stimulants, tonics and food met with greatest favor in the philosophy of one party, while expectant patience, with nature's own efforts, seemed to satisfy the other. Did we have a change of type in disease, or that other transformation? Neither; but just then new light shone upon the scene—pathological inquiry solved the doubt, as much as might be, for the time, and onward in its progress to certitude, must present medicine in a more scientific phase. Certitude, thanks to workers in every part of the universe, is being eagerly sought. The laboratory, the microscope, the test-tube, find interpretation for doubt or uncertainty in hidden or obscure disease; the dog, the cat, the rabbit, and the frog, are made to contribute their quota to this fast-acquiring certainty; and, although one can see, read and hear as of old, that satisfaction is not proved to all the dissidents, but add their mite to that truth that sooner or later must prevail, when medicine shall keep its "promise to the sick."

To physiological research, more than to any other, are we indebted for some of the greatest triumphs in modern medicine, reflex action, cerebral localization—marvels of discovery, utility, and application. Ere long the cause of, or the organs involved in, the production of fever will have been discovered, when no longer we shall grope in the dark, nor be governed by contradictory opinions, but shall have a clear and valid elucidation of the facts observed, through our knowledge of the physiological functions invaded by that uncertain or unknown quantity called disease. Pyrexia and hyper-pyrexia will be explained by laws proven to belong to the ganglionic system of nerves in a state of pathological perturbation; and this disturbance will be accounted for by recognizing the germ theory of disease, by consenting to a *casus belli* in the economy between the different tribes of bacteria (and thus prove the cogency of the Darwinian theory of the survival of the fittest), or by accepting the fact that the body, in a susceptible state, from a great variety of causes, receives, through air or food, or generates within itself, some septic poison that interferes with the normal function of some special ganglionic center, which readily resents the encroachment upon its duty, and repels with force of cold and heat the direful enemy. This, and much more, physiological experiment will bring about, and make more certain and useful that actively worked field of pathological research which has for its object a more definite explanation of the origin, propagation, and mode of communication of disease. In this effort, no fault can be found, though laborers are many, work so extensive, and the lines of investigation, as well as subjects, widely different—not, therefore, the less interesting, on the whole. The value of this ardently cultivated department of medicine would, however, be greatly enhanced if there were a better consensus of opinion among the many workers. It would be a guide of greater certitude at the bedside, where the judgment should be without doubt, the action consequently wise, and the result satisfactory.

Notwithstanding that the data of physiological experiment indicate a great advance upon former times, being in possession of so many means of research not heretofore known, much greater results are expected of it in the future. It *must* establish the existence or non-existence of inhibitory centers, of trophic centers, of heat and other centers, of vaso-motor, constrictor-dilator nerves, exercising or not the functions now attributed to them through these or other centers of the cerebro-spinal system. So, too, pathology, which has so exalted an errand to perform, will be, *must* be, cultivated with a view to establish that consensus of opinion referred to, and so much to be desired. It is not enough that the school of biologists and pathologists asserts that all communicable or contagious diseases are due to bacteria and allied organisms—it must be proved. It is not enough that great and talented cultivators in this field have labored earnestly, and, mayhap, honestly, to discover the true theory of pathogenesis; that they have solved the difference between spore and germ; and have given us a classification of micro-organisms that, to themselves, may be of easy comprehension, but, to the large proportion of practical men, of no value, because not proving such micro-organisms to be the source from which disease arises. Yet how loudly is it proclaimed in the general cry for antiseptics.

Assuming that this inquiry has been rather to ascertain the multiplicity of the tribes of cocci, bacilli, bacteria, etc., than to elucidate with any useful certainty the cure or prevention of disease, is it not necessary, is it not imperative, that they shall give us laws governing their doctrines that we can utilize? Of what avail that we recognize the tubercle bacillus, the cholera microbe, *et genus omne*, if we can make no use of this knowledge to keep our promise to the sick? An innocent swelling is threatened with some malignant metabosis: has pathological analysis pointed to the therapeutics of prevention? Here is the key to what is required of pathological investigation—to the end that the *materia medica* may subserve its purposes in the cure of disease, the *summa* of our calling.

Where does therapeutics stand in its relation to advancing time? The materia medica has undergone great changes; chemistry and pharmacy have altered the crudeness of the medicines of by-gone days; and pharmacology, though of recent growth, will displace empirical methods which, in all time, even to this present, have him who used them prudently most faithfully served, and supply instead the rational.

When, as we know, how amyl nitrite the pangs of angina pectoris allays, so shall we know how digitalis regulates a weak and irregularly beating heart; how strychnia to the nervous system stimulus and tone doth bring; and phosphorus waste of its elements restores. Quinine—we know it ague cures, but how? Not yet a satisfactory explanation, though guesses are innumerable. Iodide of potassium and mercury, of deobstruments most potent—upon what principle and through what source we should positively know. The discovery of ether and chloroform might be considered chief among the evidences of advancing knowledge in this department, for, as boon to man and beast, no greater can be found; and so with many more. There are diseases, however, of which we learn a great deal through pathological histology, but know nothing of their appropriate medicines. This opprobrium of our art must disappear before the more accurate knowledge obtained through pharmacology, which now, like the other branches of medical science mentioned, is being cultivated with great hope of reaching a precision that will be hailed with delight by the practicing physician.

Having indicated a general progress in physiology, pathology, and therapeutics, from earliest time to the present, and pointed to the urgent necessity of its continuance, I might be expected to suggest a means to the fullest possible accomplishment of our desires. It is summed up in a few words: Select from the many who would study medicine those already trained in letters and the arts collateral. Inspire them with a deep reverence for their profession and a keen sense of its awful responsibilities, and impart to them, then, through fitted teachers, and for five long years, such knowledge of medicine as will enable them to further its highest development, so that "as agriculture, to those who are in health, holds out the expectation of aliment, medicine shall promise to the sick a recovery from disease," and keep the promise.

